# Novel Roles of Follistatin/Myostatin in Transforming Growth Factor-β Signaling and Adipose Browning: Potential for Therapeutic Intervention in Obesity Related Metabolic Disorders

**DOI:** 10.3389/fendo.2021.653179

**Published:** 2021-04-09

**Authors:** Shehla Pervin, Srinivasa T. Reddy, Rajan Singh

**Affiliations:** ^1^ Department of Obstetrics and Gynecology, David Geffen School of Medicine at University of California Los Angeles (UCLA), Los Angeles, CA, United States; ^2^ Division of Endocrinology and Metabolism, Charles R. Drew University of Medicine and Science, Los Angeles, CA, United States; ^3^ Department of Molecular and Medical Pharmacology, David Geffen School of Medicine at UCLA, Los Angeles, CA, United States; ^4^ Department of Medicine, Division of Cardiology, David Geffen School of Medicine, University of California Los Angeles, Los Angeles, CA, United States; ^5^ Department of Endocrinology, Men’s Health: Aging and Metabolism, Brigham and Women’s Hospital, Boston, MA, United States

**Keywords:** follistatin, myostatin, obesity, transforming growth Factor β, UCP1, adipose tissue

## Abstract

Obesity is a global health problem and a major risk factor for several metabolic conditions including dyslipidemia, diabetes, insulin resistance and cardiovascular diseases. Obesity develops from chronic imbalance between energy intake and energy expenditure. Stimulation of cellular energy burning process has the potential to dissipate excess calories in the form of heat *via* the activation of uncoupling protein-1 (UCP1) in white and brown adipose tissues. Recent studies have shown that *activation* of transforming growth factor-β (TGF-β) signaling pathway significantly contributes to the development of obesity, and blockade or inhibition is reported to protect from obesity by promoting white adipose browning and increasing mitochondrial biogenesis. Identification of novel compounds that activate beige/brown adipose characteristics to burn surplus calories and reduce excess storage of fat are actively sought in the fight against obesity. In this review, we present recent developments in our understanding of key modulators of TGF-β signaling pathways including follistatin (FST) and myostatin (MST) in regulating adipose browning and brown adipose mass and activity. While MST is a key ligand for TGF-β family, FST can bind and regulate biological activity of several TGF-β superfamily members including activins, bone morphogenic proteins (BMP) and inhibins. Here, we review the literature supporting the critical roles for FST, MST and other proteins in modulating TGF-β signaling to influence beige and brown adipose characteristics. We further review the potential therapeutic utility of FST for the treatment of obesity and related metabolic disorders.

## Introduction

The obesity epidemic significantly affects every region and demographical group worldwide with no signs of abatement. Obesity substantially increases the risk for several chronic diseases including cardiovascular diseases, fatty liver, diabetes, insulin resistance and cancer. It has been estimated that by 2030 approximately 2.16 billion individuals will be overweight and 1.12 billion individuals will be obese as defined by body mass index (BMI) of 30 or higher (1). The economic impact of obesity and its related complications on United States has been estimated between 4-8% of gross domestic product and comparable to 2018 defense budget ($643 billion) and Medicare ($588 billion) (2), and significantly impacts low-income and economically disadvantaged populations. The strategies, to date, to combat the obesity epidemic have not been successful and there is an unmet need for the development of novel therapies to prevent and treat obesity and related metabolic complications. As obesity develops from surplus energy stored in adipose tissues, therapeutic approaches to reduce energy intake, increase energy expenditure, or both would provide attractive avenues for the fight against obesity and related diseases. Although thermogenic adipocytes and their precursors are composed of various distinct cell populations (3, 4), adipose tissue mass is composed mostly of white adipose tissue (WAT) and brown adipose tissue (BAT), which metabolically play opposing roles in regulating energy balance.

Recent clinical cross-sectional studies using [18F] FDG-PET/CT, suggest a clear decline in BAT activity and mass during aging that coincides with the development of obesity and insulin resistance. Several laboratories have presented evidence for expression of the thermogenic molecule UCP1 as well as its energy dissipating capacity in human BAT and contribute towards improved metabolic profiles (5–7). WAT, which is specialized for storage of energy could be manipulated *via* genetic or pharmacological means to promote browning. Such browning, also called as “beige” or “brite” (brown in white), is associated with increased expression of mitochondrial uncoupling protein-1 (UCP1) expression in response to external stimuli including chronic cold exposure, treatment with β-adrenergic agonists CL 316,243, exercise, and endocrine factors (8, 9). This type of adipose browning is also associated with increased thermogenic capacity of the cells since activation of UCP1 that uncouples mitochondrial respiration from ATP production provides significant metabolic benefits that are comparable to BAT (8, 9). Experimental mice with selective ablation of beige adipose cells are prone to obesity and metabolic dysfunction probably by reducing lipogenic capacity and energy expenditure as well as by modulating the inflammatory environment inside the WAT (10). Overexpression of Prdm16 (PR-domain containing 16) resulted in abundant beige adipocytes in subcutaneous adipose depots, associated with significantly increased energy expenditure and were resistant to weight gain in response to a high fat diet (HFD) (11). Similarly, CRISPR/CAS9-mediated reconstitution of UCP1 in WAT of pigs led to significantly decreased fat mass and improved energy expenditure (12), suggesting the therapeutic potential of modulating WAT phenotype by activation of key thermogenic genes for fighting obesity and metabolic syndrome. While implementation of findings from animal studies remains challenging to apply in humans, such studies have yielded novel insights into the molecular mechanisms underlying thermogenic regulation of brown and beige adipocytes and highlight their ability to reduce obesity and related metabolic disorders. Thus, further studies directed to translate the proofs of concept generated in animal models are crucial.

Transforming growth factor-beta (TGF-β) signaling has been shown to regulate glucose and energy homeostasis (13). TGF-β levels are reported to increase with adiposity in overweight (BMI between 25-29.9 kg/m^2^) and obese (BMI ≥ 30 kg/m^2^) subjects compared to the normal subjects with BMI less than 24.9 kg/m^2^), and systemic blockade of TGF-β/SMAD3 signaling resulted in protection against diet-induced obesity in experimental mice (13). This effect was associated with acquisition of energy dissipating brown adipocyte phenotype in WAT. In this review, we will discuss the evidence for the novel role of FST, MST and other related proteins in modulating TGF-β signaling and adipocyte browning to explore possible therapeutic avenues for the treatment of obesity and associated metabolic disorders.

## Beige and Brown Adipocyte Developmental Origin and Molecular Signatures

White, beige and brown adipocytes are three major types of adipocytes that have distinctly different fat morphology and differ in their developmental origin as well as function. During embryogenesis, BAT development precedes the formation of WAT, where it primarily contributes to non-shivering thermogenesis and maintain body temperature in newborns. Interscapular BAT mainly contributes to the temperature regulation during early stages of life and its levels slowly regress with age (14–16). Lineage-tracing studies demonstrated that classical brown adipocytes present in BAT depots originate from a sub-population of dermomyotome expressing specific transcription factors, including Pax7, engrailed 1, and Myf5 (17–20). Previously, these Myf5-expressing (Myf5+) precursors were assumed to be present exclusively in skeletal muscle precursors and absent in both in WAT and beige adipocytes (20, 21). Beige adipocytes present in the inguinal white adipose depots are reported to be derived from Myf5 negative (Myf5-) precursor pool (22). However, more recent lineage tracing studies have identified subsets of white adipocytes that are derived from both Myf5+ and Myf5- precursors (23–25), and beige adipocytes are derived from progenitor populations expressing Sma, Myh11 (a selective marker for smooth muscle cells), platelet-derived growth factor receptor (PDGFR)-α, or PDGFR-β in mice (26–29). Retinoic acid (RA)-induced adipose browning in endothelial cells and capillaries has also been reported *via* the activation of vascular endothelial growth factor (VEGF) A/VEGFR2 signaling that facilitate PDGFR-α-expressing adipocyte precursors (18). Beige cells could also appear as a result of trans-differentiation of mature white adipocytes (15). Trans-differentiation of beige adipocytes to WAT has been reported during warm adaptations and aging (30, 31). Collectively, the above studies strongly suggest that beige adipocytes that emerge in WAT depots appear to have multiple origins compared to the brown adipocytes.

PGC-1α is a master regulator of adaptive thermogenesis that binds to PPAR-γ and coactivates PPAR-γ to stimulate the transcription of genes involved in the brown adipocyte differentiation process and acquisition of morphological and molecular features of brown and beige fat (32). PGC-1α expression is rapidly induced by cold exposure that turns on several key components of the adaptive thermogenic program including fatty acid oxidation, mitochondrial biogenesis, and increased oxygen consumption (33). The transcriptional factor PR domain zinc finger 16 (PRDM16) is selectively expressed in brown/beige compared to the visceral white fat cells and plays an important role in controlling the differentiation-linked brown adipose/skeletal muscle fate determination and gene expression program (34). Gain and loss-of-function studies of PRDM16 in various cell systems have clearly established its major role in brown adipose/skeletal muscle cell fate determination (35). Using analysis of clonal cell lines, Wu et al. also suggested that beige and brown adipose cells express related but distinctly different gene expression profiles (36). Beige cells are selectively enriched in Tmem26, Tbx1, and CD137 expression (36). The same study identified additional beige selective genes including Ear2, CD40, Sp100, Klh113, and Slc27a from interscapular BAT and inguinal fat. Wang et al. identified early B-cell factor 2 (Ebf2) as one of the most selective markers for brown and beige adipogenic precursor cells (37). More beige-selective genes including *HoxC8*, *HoxC9*, *Cited1*, and *Shox2* were identified using molecular profiling of human BAT (38, 39). On the other hand, epithelial V-like antigen (*Eva1*), *Lhx1*, *Zic1*, and *Epsti1* are selectively expressed in classical brown adipocytes (36–41). Additionally, *Ebf3*, *Pdk4*, *Fbxo31*, *Oplah*, and *Hsbp7* were also found to be highly enriched in interscapular BAT of 129SVE mice (36). Ussar et al. have reported few selective cell surface markers for white, beige and brown adipocytes that could provide unique tools to identify various adipocyte populations in both humans and rodents and potentially target them for therapy *in vivo* (42). The authors identified amino acid transporter *Asc1*, encoded by the SLC7A10 gene as a white adipocyte-specific cell surface protein, which was barely expressed in brown adipocytes (42). Expression level of purigenic receptor P2RX5, part of a seven-member family of ATP gated ion channels, was highest in brown adipocytes. Proton coupled amino acid transporter PAT2, another cell surface protein, show highest specificity for adipose tissue among all three markers identified in this study with significantly higher expression in brown fat compared to white fat. Better understanding of the gene expression pattern of such adipose-specific cell surface markers should provide novel tools to selectively mark and access intact white and brown adipocytes and could be used for diagnostic and therapeutic purposes. Analysis of microRNA (miRNA) between beige and brown fat have provided clear differences in their expression profile. Several miRNAs including miRNA-30, miRNA-182, and miRNA-203 are reported to positively regulate both beige and brown adipocytes (43, 44). On the other hand, miRNA-27 and miRNA-34a negatively regulator beige and brown adipogenesis (45, 46). Recent studies have also highlighted some specific miRNAs including miRNA-196b and miRNA-26 that positively and negatively regulate beige adipogenesis respectively (47, 48).

## Transforming Growth Factor-Beta (TGF-β), Adipose Browning and Obesity

The TGF-β superfamily consists of several members including TGFβ1, TGFβ2, and TGFβ3, bone morphogenetic proteins (BMPs), growth differentiation factors (GDFs), and activins that regulate diverse biological processes during embryogenesis, adult tissue homeostasis, and function of several cell types including adipocytes (49, 50). The pleiotropic effects of TGF-β/Smad3 signaling on cell metabolism and energy homeostasis plays an important part in the progression of obesity-linked diabetes; these include adipocyte differentiation, adipose browning, inflammation and regulation of insulin signaling amongst others. Members of TGF-β superfamily transmit their signals *via* dual serine/threonine kinase receptors and transcription factors called Smads. Recent studies have clearly established an essential role of TGF-β/Smad3 signaling in the pathogenesis of obesity and type 2 diabetes. Elevated levels of TGF-β has been reported in mice and human adipose tissue during hypertension and other cardiovascular diseases as well as in morbid obesity and diabetic neuropathy (51–53). Increased TGF-β levels have also been associated with a higher risk for type 2 diabetes in a prospective case-cohort study (54). Perry et al. identified Samd3 gene in a type2 diabetes genome-wide association study (55). Smad3 is known to bind to the PGC-1α promoter to repress its transcription (13). As PGC-1α is an important transcriptional coactivator for UCP1 gene induction, mitochondrial biogenesis, and fatty acid oxidation, it is not surprising that TGF-β/Smad3 signaling would inhibit beige/brown adipocyte differentiation and their thermogenic action. The discovery of TGF-β/Smad3 signaling as novel modifiers of beige adipocyte phenotype and metabolic characteristics by Yadav et al. has opened therapeutic avenues for identifying potent inhibitors of this signaling pathway for the treatment of obesity related complications (13). The authors observed significant positive correlation between TGF-β1 levels and adiposity in both rodents and human subjects. Smad3^−/−^ mice displayed protection against diet-induced obesity and related metabolic syndromes. These effects were associated with significant induction of white to brown phenotype and increased mitochondrial biogenesis. Examination of a group of nondiabetic human subjects from diverse ethnic groups, the authors found direct relationship between circulating TGF-β1 levels and BMI, fat mass, and oxygen consumption. The same group assessed the effect of blocking TGF-β/Smad3 signaling in two well-characterized mouse models of obesity and type 2 diabetes. Treatment with anti-TGF-β neutralizing antibody 1D11 resulted in significantly reduced body weight, improved glucose and insulin tolerance, as well as fasting glucose and insulin levels. These beneficial effects were associated with elevated expression of BAT and mitochondria-specific proteins including UCP1, COX-1 and PGC-1α as well as decreased phosphorylation of Smad3 in white adipose tissues. Such links between TGF-β signaling and mitochondrial energy metabolism pathway have also been reported by other laboratories (56). In addition, several studies have demonstrated extensive interaction between TGF-β and key energy sensors including adenosine monophosphate protein kinase (AMPK) and sirtuin family members (57, 58). Inhibition of activin receptor IIB (ActRIIB) responsible for integrating actions of TGF-β ligands promotes differentiation of primary brown adipocytes *in-vitro* and increases brown fat mass, but not white fat mass in mouse (59). Furthermore, inhibition of ActRIIB *via* a decoy receptor containing extracellular domain of ActRIIB fused with human Fc (ActRIIB-Fc) resulted in suppression of diet-induced obesity and related metabolic complications in mice (60). This blockade of ActRIIB was associated with increased browning and robust upregulation of UCP1 and PGC-1α expression in the epididymal white adipose fat and led to increased energy expenditure under ambient or cold temperature. Gene signature induced as result of ActRIIB inhibition, displayed an interesting similarity with PGC-1α overexpression *in-vivo*. Combined together, these studies provide significant insights into the role of TGF-β signaling in suppressing adipose browning program within white fat tissues in both mouse models and human subjects, suggesting that blockade of TGF-β activity could serve as an effective treatment strategy for obesity and diabetes.

Since bone morphogenic proteins (BMPs) belong to the same superfamily of growth factors as TGF-β, and regulate various aspects of white and brown adipocyte differentiation, we discussed below briefly their biological functions in modulating adipose tissue functions (61–66). BMP4 has been shown to promote differentiation of human adipose stem cells into beige adipocytes (61, 62). BMP4 overexpressing transgenic mice display reduced adiposity, improved insulin sensitivity, and induction of brown adipocytes within inguinal subcutaneous fat depots (63, 64). Interestingly, these transgenic mice display decreased expression of brown adipocyte markers including UCP1 and PGC-1α in the BAT (62). In spite of reduced BAT activity, these BMP4 overexpressing mice are protected from diet-induced obesity and insulin resistance perhaps due to increased WAT browning (63). It, therefore, appears that BMP4 may have opposite effects on the development of brown adipocytes in BAT and beige adipocytes in WAT *in-vivo*. BMP7 promotes the commitment of mesenchymal progenitor cells to a brown adipocyte lineage while it prevents osteogenesis by inhibiting the expression of runt-related transcription factor 2 (*Runx2*) (67). In C3H/10T1/2 cells, pretreatment with BMP7 results in brown adipogenesis with lipid accrual and expression of *Ucp1* (67). Tail vein injection of adenovirus expressing BMP7 increases BAT, without affecting the mass of WAT (67). Although BMP7 increases Prdm16 and Ucp1 expression in brown adipose, there are no changes in the expression of genes involved in energy metabolism in white adipose, muscle, or liver. The increase in BAT mass results in increased energy expenditure, higher basal body temperature, and decreased body weight attributes that clearly link BMP7 signaling to energy balance. BMP7 knockout mice show significant reduction of brown fat mass (67). Conversely, adenoviral-mediated expression of BMP7 in mice results in significant increase in brown fat mass, increased energy expenditure and reduction in weight gain and subcutaneous implantation of BMP7-treated MSCs into athymic nude mice results in ectopic brown adipose tissue formation (67). BMP8b promotes brown adipose tissue thermogenesis through both central and peripheral actions (65). This thermogenic effect of BMP8a is observed only in female mice and is thought to be mediated by estrogens (66). The molecular mechanisms responsible for such differential regulation of WAT, beige and BAT by various BMP members remains largely unknown.

## Myostatin, Irisin, Adipose Browning and Energy Metabolism

Myostatin (MST), also referred to as growth and differentiation factor 8 (GDF8), is a member of TGF-β superfamily. MST is synthesized as a precursor protein, which consists of a N-terminal propeptide domain that contains the signal sequence and a C-terminal domain that forms a disulfide-linked dimer and functions as the active ligand (68). MST requires release from the propeptide to be biologically active (69). MST binding to ActRIIB leads to the phosphorylation of Smad3  (70). Phosphorylated Smad3 can bind other Smad proteins and these complexes translocate into the nucleus, where they regulate the transcription of target genes  (70). It is mainly expressed in skeletal muscle but is also detectable in cardiac muscle, blood, and to a limited extent in adipose cells. MST is known as the potent negative regulator of muscle mass as inactivation of *Mst* gene significantly accelerates muscle growth in cattle, sheep, fish and humans (71–75).

Recent studies from several laboratories have provided conclusive evidence that the effect of MST extends beyond its role in skeletal muscle, and it plays a significant role in the regulation of body fat and overall energy metabolism. Mst-knockout (*Mst*-KO) mice show significantly increased muscle mass, decreased fat mass, improved insulin sensitivity and resistance to diet-induced obesity (76, 77). On the other hand, overexpression of MST in mice has been shown to promote catabolic conditions and result in muscle wasting and cause insulin resistance (78). Since MST is expressed in very low amounts in fat tissues, it is not clear how lack of MST can suppress fat accumulation in *Mst*-KO mice. Significantly increased energy expenditure and leptin sensitivity was observed in *Mst*-KO mice that could potentially explain reduced fat mass in these mice when compared to the WT mice (79). In primary cultures of mouse preadipocyte cells, Kim et al. reported decreased expression of key thermogenic genes *Ucp1*, *Prdm16*, and *Pgc-1a* and significant inhibition of brown adipogenic differentiation following treatment of the cells with recombinant MST protein (80). Using differentiating primary cultures of mouse embryonic fibroblast (MEF) isolated from *Mst*-KO and WT embryos, Braga et al. reported significant upregulation of key thermogenic markers in differentiating cultures of *Mst*-KO group compared to the WT group (81). In the same study, treatment with recombinant MST protein led to a significant decrease in Oil-Red O stained adipocytes and expression of key thermogenic genes in both WT and Mst-KO groups. Comparative analyses of epididymal (Epi) and subcutaneous (SC) adipose tissues isolated from WT and *Mst*-KO mice show clear induction of thermogenic proteins including UCP1, and PRDM16 along with C/EBPα. Gene expression analyses further confirmed significant upregulation of key adipogenic differentiation markers *Cebpα* and *Pparγ*, as well as key thermogenic genes including *Prdm16, Ucp1, Bmp7, PGC-1a/b* and *Cidea*, suggesting that loss of MST significantly promotes brown adipose-related markers in two main adipose depots in *Mst*-KO mice (81). Similar comparative analyses of muscle tissues from androgen-dependent (levator ani, LA) and independent (gastrocnemius, Gas) muscle tissues show upregulation of UCP1 and PRDM16 protein and several genes involved in the regulation of overall thermogenic program. These combined in-vitro and *in-vivo* approaches using differentiating MEF cultures, as well as *Mst*-KO and their WT littermates show that MST inhibition could not only promote white adipocyte browning in adipose depots but could also promote the conversion of inter-muscular white adipocytes into beige/brown adipocytes. Furthermore, protein expression analysis of energy-sensing adenosine monophosphate (AMP)-activated protein kinase (AMPK), a critical regulator of mitochondrial biogenesis that controls energy metabolism by acting in co-ordination with NAD+-dependent type III deacetylase sirtuin1 (Sirt1) was found to be significantly upregulated in differentiating *Mst*-KO MEF primary cultures compared to the WT group (81). Protein expression of adiponectin, a key protein secreted from adipocytes and regulator of adipocyte energy metabolism was also found to be upregulated in differentiating MEF cultures isolated from Mst-KO group compared to the WT (81). Adiponectin is reported to limit triglyceride (TG) accumulation in liver (82), increase glucose clearance and improve hepatic insulin action in adiponectin transgenic mice (82, 83). Zhang et al. also reported that inhibition of MST leads to increased skeletal muscle mass, slows down fat accumulation, lowers body weight and circulating levels of triacylglycerol in mice on high-fat diet (84). The authors reported that white adipose tissue of *Mst*-KO mouse express significantly higher levels of genes involved in lipid transport, synthesis, oxidation and hydrolysis. In addition, adipose tissues isolated from *Mst*-KO mice show increased expression of UCP1 and upregulation of AMPK signaling pathway when compared to the WT mice (84). Histological analysis of WAT isolated from Mst-KO revealed BAT-like cells filled with multilocular smaller lipid droplets and immunopositive for UCP1 (81), suggesting that MST deletion induced brown-like phenotype. Genetic loss of MST has also been reported to promote white adipose browning and improve insulin sensitivity by several other laboratories (85, 86). Shan et al. performed a thorough analysis of various muscle-derived circulatory factors to identify possible mediators of adipose browning phenotype in these Mst-KO mice (86). The authors reported that skeletal muscle derived irisin (encoded by *Fndc5* gene) plays a central role in promoting adipose browning in *Mst*-KO mice by activating AMPK-PGC-1α-Fndc5 signaling, providing an interesting involvement of muscle-adipose cross talk during adipose browning (86). Dong et al. also reported the involvement of Fndc5/irisin-mediated white adipose browning and improvement in insulin signaling in Mst-KO mice (87). *Mst*-KO Meishan pigs with functional deletion of Mst show increased insulin sensitivity, adipose browning and upregulation of several browning-like gene signature including *Ucp1, Prdm16, Pgc-1α, Cidea*, *Cd137* and *Tmem26* (85). Protein expression analysis of skeletal muscle in these *Mst*-KO pigs shows significantly increased levels of insulin receptor (IR) and insulin receptor substrate (IRS). Skeletal muscle protein expression of irisin precursor protein Fndc5 as well the serum irisin levels were significantly higher in Meishan *Mst*-KO pigs compared to the WT pigs. Activation of insulin signaling pathway could not be blocked *via* inhibition of irisin in this study, suggesting possible irisin independent activation of insulin signaling in MST deficient skeletal muscle (85). Reduction of interferon regulatory factor 4 (IRF4) leads to significantly reduced exercise capacity, mitochondrial function and ribosomal protein synthesis in brown fat, an effect that was associated with induction of MST levels (88). On the other hand, overexpression of IRF4 led to significantly reduced levels of serum MST and increased exercise capacity in muscle. IRF4 was shown to physically interact with PGC-1α and promote the thermogenic program by upregulating the transcription of UCP1 gene and driving mitochondrial biogenesis in BAT. In addition, IRF4 levels in BAT was found to be significantly induced following cold exposure and β3-adrenergic receptor (AR) agonist (88). These findings, therefore, suggest that IRF4 is a novel inducer of overall thermogenic program with the potential to inactivate MST bioactivity. Guo et al. reported additional role of MST regulation in the development of proatherogenic dyslipidemia, insulin-mediated glucose disposal as well as protection against hepatic steatosis (89). The authors show that administration of adeno-associated virus 9 (AAV9)-mediated MST pro-peptide significantly blocked the progression of atherosclerosis and development of hepatosteatosis in LDLR^-/-^ mice on western diet. In this study, the beneficial effects of both *Mst* genetic ablation as well as its inactivation by MST pro-peptide were attributed to result from the enlarged muscle mass although the authors did not study its effect on adipose browning and brown fat activation. Several laboratories provided compelling evidence to support the notion that brown fat activation could reduce hypercholesterolemia and elicit protection form atherosclerosis development (90–92). Most recently, Pydi et al. demonstrated that increased plasma MST levels in mice lacking β-arrestin 1 (barr1) (adipo-baar1-KO) led to impaired insulin signaling in multiple peripheral tissues (93). On the other hand, overexpression of baar1 in adipo-baar1-OE mice on high fat diet displayed pronounced improvements in glucose tolerance, insulin sensitivity, and displayed significant reduction in MST levels, suggesting that overexpression of baar1 in adipocytes protects mice from obesity-associated metabolic disorders. Collectively, these data provide strong evidence that inhibition of MST could provide justification not only for increased muscle mass but could also be beneficial for the treatment of obesity and associated metabolic disorders through activation of adipose browning.

Irisin is a key myokine and adipokine that is secreted following the proteolytic cleavage of its precursor fibronectin type III domain containing protein 5 (FNDC5). Secreted irisin exerts its major action by upregulating the expression of UCP1 and promoting browning of WAT (94). Circulating levels of irisin are regulated by various factors including diet, exercise, obesity and pharmacological agents (95). Bostrom et al. first isolated irisin from muscle tissues and performed its chemical characterization (96). Following exercise stimulation and activation of transcriptional co-activator PGC-1α, FNDC5 expression levels are increased in muscle, resulting in the secretion of irisin to induce adipose browning through activation of thermogenic genes (96). These findings provided strong evidence for the beneficial role of irisin in cardiovascular, obesity, diabetes, skeletal and other diseases. Cold activation and physical activity among several other factors are known to alter the level of circulating irisin (97, 98). Plasma irisin levels are reported to increase by 65% after 3 weeks of freewheel running, while in healthy humans irisin levels double after 10 weeks of endurance exercise (96). Several studies demonstrated that irisin improves glucose homeostasis, and its circulating levels are inversely associated with liver fat content (99–101). Based on these findings, irisin was revealed as a potential new target for the treatment of metabolic diseases. However, in contrast with the above reports, several other studies question the beneficial role of irisin and in some cases even its existence (101–104). There is also a disagreement regarding the induction of FNDC5/irisin by exercise (105, 106), and its association with markers of glucose and lipid homeostasis disturbance in obesity and metabolic syndrome (107–110). Such controversies could be explained by the fact that irisin levels increase only when muscle ATP concentration decreased in absence of physical activity during sedentary lifestyle (105). Perez-Sotelo et al. reported decreased browning capacity and increased adipogenesis of differentiating adipocytes by blocking adipose endogenous expression of FNDC5 (111). The authors reported that incubation of normal adipocytes with secreted factors from the WAT of obese patients resulted in significant reduction of FNDC5, PGC-1α and UCP1 expression. Irisin is also reported to influences glucose metabolism in skeletal muscle (112) and myocytes in-vitro *via* increased oxidative phosphorylation, mitochondrial biogenesis and upregulation of various genes involved in glucose transport as well as in mitochondrial uncoupling (113). Furthermore, exogenous FNDC5 induces UCP1 expression in subcutaneous white adipocytes in animal models, and FNDC5 overexpression in the liver prevented diet-induced weight gain, metabolic disturbances, and stimulation of oxygen consumption (114). Irisin administration was also found to increase the secretion of glycerol and decrease lipid accumulation *via* regulating the expression of hormone-sensitive lipase (HSL), adipose triglyceride lipase (ATGL) and fatty acid-binding protein 4 (FABP4) (115). Moreover, irisin was found to inhibit hepatic cholesterol synthesis through AMPK-SREBP2 signaling (116) in addition to its ability to lower plasma glucose levels and altered food intake in streptozotocin-induced diabetes mellitus model (117). Subcutaneous perfusion of irisin resulted in significantly increased energy expenditure, reduced hyperlipidemia and hyperglycemia, and improved insulin resistance (118). These beneficial effects of irisin were mediated *via* upregulation of cAMP/PKA/HSL-perilipin pathway (118). In a recent report, Li et al. provided supporting evidence for a critical role of irisin in mediating Fst-induced browning (119). They reported that Fst injection promoted increased secretion of irisin from the subcutaneous fat depots *via* AMPK-PGC1-α-irisin mediated signaling during adipose browning. In cardiomyocyte H9C2 cells, recombinant irisin (r-irisin) activated PI3K/AKT pathway, induced intracellular Ca^2+^ signaling, and increased cellular oxygen consumption (120). In primary adipocytes and 3T3-L1 cells, r-irisin significantly increased the expression levels of key thermogenic genes including *Ucp-1*, *Pgc-1a*, *Cox7a*, *Ebf3*, and *Elovl3* and phosphorylated forms of p38 MAPK and ERK1/2. Pharmacological inhibition of p38 MAPK and ERK1/2 phosphorylation significantly lowered irisin-induced UCP-1 expression (94). Collectively, these studies provide novel beneficial role of irisin in regulating key metabolic parameters associated with perturbed lipid, cholesterol and energy metabolism.

## Follistatin

### Follistatin and Follistatin-Like Proteins

Follistatin (FST) was initially identified as component of the follicular fluid capable of inhibiting follicle-stimulating hormone (FSH) (121). FST is a monomeric glycosylated protein that binds and neutralizes activins with high affinity and neutralizes their bioactivity (121). FST also binds with lower affinity to several other members of the TGF-β superfamily including MST and BMPs 2, 5, 7, and 8 (122–125). These reports highlight the potential for FST to modulate the biological activities of several TGFβ superfamily, particularly at higher concentrations. Two variants of FST are generated through alternate splicing at the C-terminus of the common precursor gene (126). A third isoform of approximately 300-303 amino acids (FST300 or FST303) is also reported to be produced by proteolytic cleavage of the C-terminus of FST315 (127). The shorter isoform FST288 is capable of binding heparin-sulfated proteoglycans on the cell surface with high affinity. The longer isoform FST315 is localized primarily in the circulation and has reduced affinity for heparin as a result of masking of the heparin-binding site at the C-terminal (121). Another molecule related to FST, called follistatin-like 3 (FSTL3) has been identified (128). This protein lacks the heparin-binding sequence, but similar to Fst, it binds activin A with high affinity and activin B with relatively lower affinity (129). FSTL3 is relatively less effective in blocking endogenous activin A in various cells (130). It is, therefore, possible that the ability of FST to bind to the proteoglycan cell surface could be important for potent inhibition of activin A action. While Fst is expressed in several tissues including ovary, pituitary, muscle and adipose tissues, FSTL3 is distributed predominantly in testis, placenta, heart and pancreas (131). Moreover, unlike Fst, FSTL3 is located in the nucleus, though it is also secreted at a relatively slower rate (131). Based on the tissue distribution, subcellular localization and intracellular transport pattern of FST and FSTL3, it is evident that they are not functionally redundant. Glycosylation of these core proteins produces a number of protein variants ranging in size from 31 to 42 kDa in size. Human FST is glycosylated at two specific sites, but point mutation of these sites does not change the affinity of FS315 for activin A (132). It is important to note that FSTL3 along with GASP1 and MST-propeptide binds with MST in circulation, suggesting that FST might not be the sole physiological regulator of MST *in-vivo* (133–135).

### Follistatin and Muscle Mass

Matzuk et al. elegantly assessed the role of FST in regulating muscle mass and reported that FST loss-of-function mutant (*Fst*-KO) mice show decreased diaphragm and intercostal muscles and die within hours of birth (136). Based on the role of MST in being the most potent negative regulator of muscle mass to date and its inhibition by FST, Amthor et al. explored the role of possible interaction between FST and MST during chick development using yeast and mammalian two-hybrid system (123). The authors demonstrated that FST and MST interact directly with a high affinity of 5.84 × 10^−10^ M, and are expressed in the overlapping domains during muscle development. Moreover, MST-induced decrease in the expression levels of key myogenic proteins Pax3 and MyoD was significantly blocked in the presence of FST, suggesting an important role of FST in antagonizing the inhibitory effect on muscle development. Subsequently, it was reported that FST-induced muscle hypertrophy was associated inhibition of both MST and activin A and induction of satellite cell proliferation (137). Fst gene delivery of AAV1-FST344 in normal and dystrophic mice as well as in non-human primates led to significant increase in muscle mass and strength (138, 139). Transgenic expression of *Fst* in mdx mice, a popular model for Duchenne muscular dystrophy (DMD), showed amelioration of dystrophic pathology and increase in skeletal muscle mass (140). Interestingly, in a gene therapy trial Mendell et al. demonstrated beneficial effects of FST344 direct delivery into intramuscular quadriceps in patients suffering from Becker Muscular Dystrophy without any apparent side effects (141). Initially, FST was identified as a direct downstream target of testosterone action during its pro-myogenic action in both mouse models (142) and cell-culture studies (143). Protein and gene expression of FST was significantly upregulated in mouse mesenchymal pluripotent C3H 10T1/2 cells following testosterone treatment (142). This upregulation of FST was associated with a parallel increase in key myogenic markers MyoD and myosin heavy chain (MHC) II proteins, and co-treatment of the testosterone treated C3H 10T1/2 cells with anti-FST antibody abolished the myogenic action of testosterone. Furthermore, castration-induced decrease in FST expression was normalized to basal levels following testosterone supplementation, suggesting an intermediate role of FST in mediating testosterone’s promyogenic action on muscle mass (142). Subsequently, Braga et al. reported for the first time that FST is expressed in primary cultures of muscle satellite cells and respond to the myogenic action of testosterone (143). FST significantly antagonized the TGF-β-induced inhibition of MHC II expression and phosphorylation of Smad2/3 in satellite cells (143). Combined together, these findings provide conclusive support for a central role of FST in promoting muscle mass and function, and its potential therapeutic use for the treatment of muscle wasting cachexic conditions often associated with aging, HIV, and cancer.

### Follistatin and Adipose Browning

Although the role of FST in regulating skeletal muscle mass has been supported by abundant literature, its potential role in lipid metabolism has not been thoroughly investigated. Based on the established role of FST in inhibiting TGF-β/MST signaling pathway known to inhibit adipose browning and thermogenic program, it is logical to hypothesize that FST may promote brown adipose characteristics and favorably alter overall lipid and energy metabolism (144). Since both skeletal muscle and brown adipose tissue share common Myf5+ precursor population, there is a possibility that severe musculoskeletal defects and death of *Fst*-KO newborn pups could also be complicated by their concomitant decrease of BAT mass and activity, resulting in their inability to maintain proper body temperature especially during the early neonatal life. Braga et al. provided the first evidence for a potential role of FST in regulating brown adipose metabolic characteristics and thermogenesis (144). First insight regarding a direct role for FST in adipose tissues was obtained from analysis of Fst gene expression of a tissue panel from C57BL6/J mice that included WAT (inguinal subcutaneous and epididymal) and BAT depots, as well as several other metabolic tissues including brain, heart, intestine, liver, skeletal muscle, and testis (144). Interestingly, Fst gene expression was highest in BAT and skeletal muscle, and at substantial levels in inguinal WAT and liver compared to other tissues where its expression was significantly low. This finding, therefore, suggested a possible novel role of FST in regulating WAT and BAT metabolic characteristics. Differentiated mouse brown preadipocyte primary cultures show significant upregulation of FST expression along with key thermogenic markers UCP1 and PRDM16, compared to the undifferentiated cells. Interestingly, *Fst* gene expression dramatically increased in mouse BAT following cold-exposure, suggesting a possible functional role of FST during brown adipocyte differentiation and regulation of thermogenesis. Comparative analysis of mouse embryonic fibroblast (MEF) primary cultures isolated from WT and Fst-KO embryos, Braga et al. demonstrated significant inhibition of key brown adipogenic markers including PRDM16, UCP1 and PGC-1α in Fst-KO differentiating MEF cultures compared to the WT group (144). Treatment of these cells with recombinant FST protein (rFST) resulted in significant upregulation of BAT-related genes and proteins in both WT and *Fst*-KO MEF differentiated cultures. Comprehensive analysis of global gene expression profile revealed lipid metabolism pathways as the most significantly altered pathways between WT and *Fst*-KO groups. Furthermore, *Fst*-KO differentiating cultures displayed significantly compromised basal mitochondrial respiration compared to the WT group. Addition of exogenous rFST protein to the *Fst*-KO cultures rescued this respiration impairment by increasing the cellular respiration. In addition, expression level of phosphorylated adenosine monophosphate (pAMPK), a key energy sensor implicated in the regulation of cellular energy balance, was significantly down regulated in *Fst*-KO compared to the WT MEFs. More recently, Li et al. further confirmed FST-induced adipose browning in high fat diet (HFD)-fed obese mice (119). The authors demonstrated that intraperitoneal injection of FST increased thermogenesis, energy expenditure and browning of subcutaneous adipose fat in mice on HFD. FST injected mice had significantly higher body temperature 37.5^0^C compared to the control group. This FST-induced thermogenesis was further confirmed by infrared imaging that demonstrated high-temperature areas in the FST injected group compared to the control group (119). In agreement with previous reports, a recent study reported that a single injection of AAV-mediated FST administration after several weeks of HFD feeding induced browning of subcutaneous WAT by upregulation of PGC-1α, PRDM16, UCP1 and beige-specific CD137, and decreased obesity-associated metabolic inflammation (145). Collectively, these data provided interesting novel insight regarding the importance of FST in modulating lipid and energy metabolism and suggest that overexpression of FST *in-vivo* may promote both beige and brown adipose tissue mass and activity.

### Molecular Targets of FST During Adipose Browning

Using follistatin transgenic (*Fst*-Tg) mice (146), Singh et al. systematically analyzed the effect of FST in both WAT and interscapular classical BAT (147). These *Fst*-Tg mice express Fst under a muscle-specific promoter and have significantly elevated (1.5 fold) circulating levels of FST as well as interscapular BAT mass (70% higher) compared to age-matched WT control mice (147). Analysis of BAT signature genes important for differentiation (*Ucp1, Prdm16, Zic1, Myf5, Lhx8*), fatty acid oxidation (*Ascl1, Fabp3, Cidea*) and mitochondrial biogenesis and function (*Pgc1α, Cox7a1, Cox8*) as well key thermogenic proteins (UCP1, PRDM16, PGC1α) were significantly upregulated in the interscapular BAT of the *Fst*-Tg mice compared to the WT mice (147). Comparative analysis of epididymal and subcutaneous WAT between *Fst*-Tg and WT mice displayed similar upregulation of key BAT-related markers. These changes observed in both WAT obtained from *Fst*-Tg mice was associated with distinct adipose browning characteristics including increased UCP1 immunostating, and upregulation of key beige-specific *Cd137* gene in both WAT depots, with greater changes observed in subcutaneous WAT compared to the epididymal WAT. These findings provided the first line of evidence that Fst promotes adipose browning in both WAT depots and increase BAT mass *in-vivo*.

Analysis of molecular targets of FST in these two adipose depots identified two distinctly different mechanisms. While FST increased phosphorylation of p38 MAPK and ERK1/2 in both WAT depots, it increased Myf5 expression in BAT of Fst-Tg mice (147). The authors also utilized in- vitro model of differentiating 3T3-L1 cultures to confirm that recombinant FST (rFST) treatment led to significant upregulation of UCP1 and beige-specific CD137 protein and beige-selective genes *Cd137*, *Tbx1*, and *Tmem26*. This rFST-induced increase in beige-selective markers in 3T3-L1 cells was also associated with concomitant increase in p38MAPK and ERK1/2 phosphorylation. Furthermore, pharmacological inhibition of their phosphorylation in these cells by either SB023580 or PD98059 resulted in abrogation of rFst-induced upregulation of UCP1 protein expression, suggesting that FST stimulates adipose browning *via* p38MAPK/ERK1/2 pathway. Since FST overexpression in *Fst*-Tg mice displayed distinctly different targets in WAT and BAT tissues, Singh et al. analyzed differential expression of key TGF-β signaling components Smad3/pSmad3 and activin receptor type IIB (Act RIIB) in adipose tissues obtained from WT and Fst-Tg mice. FST overexpression in these *Fst*-Tg mice led to significant inhibition of Smad3/pSmad3 as well as Act RIIB expression in both SC and Epi WAT as well as in BAT (147), suggesting that inhibition of Smad3 signaling may be the common upstream target of FST action that precedes phosphorylation of p38 MAPK/ERK1/2 and activation of Myf5. FST-induced inhibition of TGF-β/Smad3 signaling has also been reported previously in satellite cells and muscle tissues (142, 143). In order to further test the effect of FST overexpression on adipocyte browning in differentiating 3T3-L1 cells, Singh et al. cloned full-length mouse Fst gene in Piggyback Transposon cargo plasmid vector to perform systematic beige/brown adipose gene expression analysis following Fst overexpression (148). Comparative gene expression analysis of Fst-overexpressing 3T3-L1 *Fst* cells with the parental 3T3-L1 cells displayed significantly higher levels of follistatin protein and gene in the cells and in the cell supernatant compared to the 3T3-L1 cells (148). Expression levels of key thermogenic and several adipose browning markers including CD137, Tbx1, and Tmem26 were significantly upregulated following Fst overexpression in differentiating 3T3-L1 cells (148). **Table 1** and **Table 2** summarizes a comprehensive list of proteins and genes respectively that are influenced by increased levels of FST in various cell culture and *Fst* transgenic (*Fst*-Tg) mouse model. *Fst* overexpression also led to significant induction of p38 MAPK and ERK1/2 phosphorylation in-vitro in 3T3-L1 confirming previous findings of induced phosphorylation of these proteins in both WAT depots of *Fst*-Tg mice. Previous reports have suggested an essential role for p38MAPK in promoting cyclic-AMP-dependent activation of protein kinase A (PKA) and activation of UCP1 transcription (149, 150). Phosphorylation of p38 MAPK following stimulation of beta-adrenergic receptor (β-AR) results in phosphorylation and recruitment of ATF2 and PGC-1α to PPRE and CRF2 motifs within the UCP1 enhancer following their interactions with PPARγ and RXRα to activate the brown adipose thermogenic program (151). Phosphorylation of p38MAPK has also been shown to stimulate adipose browning *via* induction of irisin, a key myokine that can be significantly induced by exercise and PGC1-α (117). Recent data suggest that Fndc5 is also secreted from WAT (152). Since FST is known to induce irisin encoded Fndc5 gene in mouse muscle cells (86), it is possible that induced levels of irisin/Fndc5 will have contributed to increased browning *via* phosphorylation of p38 MAPK and ERK1/2 in *Fst*-Tg mice. Robust activation of FGF21/adiponectin/pAMPK signaling pathway was found in both adipose depots of *Fst*-Tg mice suggest a possible link between *Fst* overexpression and FGF21 activation (147). In order to test the possible intermediate role of β3-AR signaling during FST-induced browning, Singh et al. also tested whether treatment of β3 agonist CL316, 243 would promote BAT activation and adipose browning in *Fst*-Tg compared to the WT mice. The authors were able to show heightened response to β3-AR activation on UCP1 expression in both WAT depots and BAT tissues obtained from *Fst-*Tg mice compared to the WT mice (147).

**Table 1:** List of proteins targeted by follistatin.

Model System	Protein	Change	Reference
Mouse embryonic fibroblast (MEF) cultures in adipogenic differentiation medium; WT vs. *Fst* KO	UCP1	↓	(144)
	PRDM16	↓	(144)
	aP2	↓	(144)
	PPARγ	↓	(144)
	PGC-1α	↓	(144)
	Cyt C	↓	(144)
Interscapular brown adipose tissue (BAT); WT vs. *Fst*-Tg	UCP1	↑	(147)
	UCP2	↑	(147)
	UCP3	↑	(147)
	PRDM16	↑	(147)
	PGC-1α	↑	(147)
	AdipoQ	↑	(147)
	Myf5	↑	(147)
	pSmad3	↓	(147)
	Smad3	↓	(147)
	ActRIIB	↓	(147)
Epididymal and subcutaneous adipose tissue; WT vs. *Fst*-Tg	UCP1	↑	(147)
	UCP2	↑	(147)
	UCP3	↑	(147)
	PRDM16	↑	(147)
	PGC1α	↑	(147)
	BMP7	↑	(147)
	Glut4	↑	(147)
	CD137	↑	(147)
	pp38 MAPK	↑	(147)
	pERK1/2	↑	(147)
	pSmad3	↓	(147)
	Smad3	↓	(147)
	ActRIIB	↓	(147)
	AdipoQ	↑	(148)
	FGF21	↑	(148)
	pAMPK	↑	(148)
Differentiating 3T3-L1 cells treated with recombinant FST (rFST); Control vs. rFST	UCP1	↑	(147)
	CD137	↑	(147)
	pp38 MAPK	↑	(147)
	pERK1/2	↑	(147)
	AdipoQ	↑	(148)
	AdipoR1	↑	(148)
	FGF21	↑	(148)
	pAMPK	↑	(148)
	PGC-1α	↑	(148)
	SirT1	↑	(148)
Mouse brown adipose tissue (BAT) cells treated with rFST: Con vs. rFST	UCP1	↑	(147)
	Eva1	↑	(147)
	Myf5	↑	(147)
*Fst* overexpressing stable 3T3-L1 (3T3-L1 *Fst*) cells; 3T3-L1-*Fst* vs. 3T3-L1	UCP1	↑	(148)
	CD137	↑	(148)
	p38 MAPK	↑	(148)
	pERK1/2	↑	(148)
	COX-IV	↑	(148)
	SirT1	↑	(148)
	SirT3	↑	(148)
	AdipoQ	↑	(148)

**Table 2:** List of genes targeted by follistatin.

Model System	Gene	Change	Reference
Mouse brown preadipocyte cells treated with rFST; WT vs. rFST	*Ucp1*	↑	(144)
	*Prdm16*	↑	(144)
	*Pgc1a*	↑	(144)
	*Fabp3*	↑	(144)
Differentiating Mouse embryonic fibroblast (MEF) cultures; WT vs. *Fst* KO	*Ucp1*	↓	(144)
	*Prdm16*	↓	(144)
	*Pgc1a*	↓	(144)
	*Bmp7*	↓	(144)
	*Pgc1b*	↓	(144)
	*Cidea*	↓	(144)
	*Acsl1*	↓	(144)
	*AdipoQ*	↓	(144)
	*Agpat9*	↓	(144)
	*Cd36*	↓	(144)
	*Fabp4*	↓	(144)
	*Mup1*	↓	(144)
	*Thrsp*	↓	(144)
	*Apoa2*	↓	(144)
	*F13a1*	↓	(144)
	*G2e3*	↓	(144)
	*Gas5*	↓	(144)
	*Ifi203*	↓	(144)
	*Titin*	↑	(144)
	*Vtn*	↓	(144)
	*Hp*	↓	(144)
	*Plg*	↓	(144)
	*Atpla2*	↓	(144)
	*Saa1*	↓	(144)
	*Cps1*	↓	(144)
	*Serpine 1*	↓	(144)
Interscapular brown adipose tissue (BAT); WT vs. *Fst*-Tg	*Ucp1*	↑	(147)
	*Prdm16*	↑	(147)
	*Zic1*	↑	(147)
	*Myf5*	↑	(147)
	*Lhx8*	↑	(147)
	*Acsl1*	↑	(147)
	*Fabp3*	↑	(147)
	*Cidea*	↑	(147)
	*Pgc1a*	↑	(147)
	*Cox7a1*	↑	(147)
	*Cox8*	↑	(147)
	*Glut4*	↑	(147)
Epididymal and Subcutaneous adipose tissue; WT vs. *Fst*-Tg	*Ucp1*	↑	(147)
	*Prdm16*	↑	(147)
	*Pgc1a*	↑	(147)
	*Acsl1*	↑	(147)
	*Fabp3*	↑	(147)
	*Cidea*	↑	(147)
	*Elov3*	↑	(147)
	*Cox7a1*	↑	(147)
	*Cox8*	↑	(147)
	*Cd137*	↑	(147)
	*Fgf21*	↑ (Epi+SC)	(148)
	*Egr1*	↑ (Epi+SC)	(148)
	*c-Fos*	↑ (Epi+SC)	(148)
	*Fgfr1*	↑ (Epi)	(148)
	*Fgfr2*	↑ (SC)	(148)
	*Fgfr3*	↑ (SC)	(148)
	*Klb*	↑ (SC)	(148)
Differentiating 3T3-L1 cells treated with recombinant FST: Control vs. rFST	*Ucp1*	↑	(147)
	*Cd137*	↑	(147)
	*Tbx1*	↑	(147)
	*Tmem26*	↑	(147)
*Fst* overexpressing stable 3T3-L1 (3T3-L1-*Fst*) cells; 3T3-L1-*Fst* vs. 3T3-L1	*Ucp1*	↑	(148)
	*Cd137*	↑	(148)
	*Pgc1a*	↑	(148)
	*Fgf21*	↑	(148)
	*Tbx1*	↑	(148)
	*Tmem26*	↑	(148)
	*Ppara*	↑	(148)
	*Fasn*	↓	(148)
	*Th*	↑	(148)
	*Bmp7*	↑	(148)
	*Ptgs2*	↑	(148)
	*Cox7a1*	↑	(148)
	*Cox8b*	↑	(148)
	*Cpta*	↑	(148)
	*Mst*	↓	(148)

**Figure 1:**
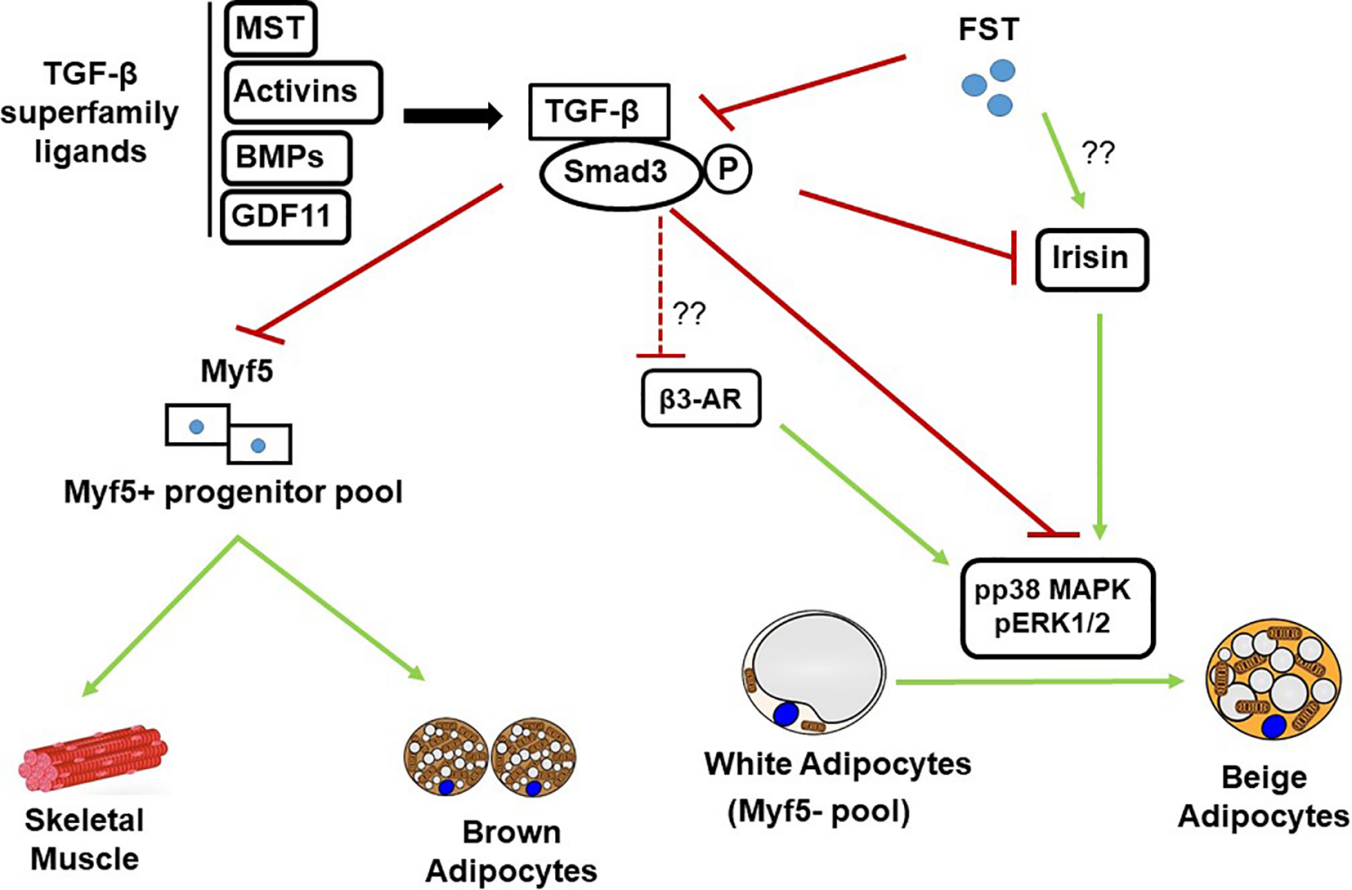
Schematic diagram showing FST modulation of TGF-β/Samd 3 signaling pathway during adipose browning. MST, Myostatin; BMPs, Bone morphogenic proteins; GDF11, Growth and differentiation factor 11; TGF-β, Transforming growth factor beta.

In order to identify the molecular targets of FST in classical brown fat, Singh et al. also analyzed the effects of exogenous rFST on differentiating mouse brown preadipocyte BAT cultures (147). rFST treatment led to significant increase in BAT-selective UCP1, Eva1, and Myf5 protein and gene expression. They also showed that siRNA-mediated knockdown of mouse Myf5 expression led to significant blockade of FST-induced UCP1 protein and gene expression and two key BAT-selective genes *Lhx8* and *Zic1*. Furthermore, *Fst*-KO embryo sections show decreased Myf5 immunostating compared to the WT, and treatment of differentiating MEF cultures derived from *Fst*-KO embryo with rFST was able to rescue Myf5 protein expression (147). Collectively, these findings obtained from differentiating BAT cells and *Fst* KO primary cultures provide strong evidence that Myf5 acts as an obligatory target of FST in promoting brown adipose characteristics. It appears however, that major action of FST on adipose browning is primarily due to the blocking of TGF-β ligands to inhibit Smad3 signaling as shown in **Figure 1**. A comprehensive list of proteins and genes targeted by FST during adipose browning are also summarized in **Tables 1** and **2** respectively.

### Genetic Manipulation of Follistatin Expression and its Relevance to Obesity Related Metabolic Diseases

As adipose tissues are the primary site of energy storage and its mobilization, activation of adipose browning has the potential to positively regulate overall systemic metabolism (153, 154). Adipose browning-induced biochemical changes are implicated in alterations of several key metabolic pathways that regulate plasma glucose levels and triglyceride metabolism in mice. Beige and brown adipose tissues consume and metabolize nutrients in a specialized way to facilitate weight loss, amelioration of insulin resistance and protection from hyperlipidemia and obesity related metabolic syndromes (155, 156). In order to assess the metabolic consequences of FST-induced adipose browning, Singh et al. performed quantitative analysis of abdominal fat volume, glucose clearance and comprehensive analysis of serum lipid profiles of *Fst*-Tg mice (148). Computerized tomography (CT) scan analysis of Fst-Tg mice revealed significantly lower percentage of abdominal fat mass and increased glucose disposal rate compared to the WT mice (148). Also, serum levels of triglycerides (TG), free fatty acid (FFA), and glucose levels were significantly lower in Fst-Tg mice compared to the WT mice without any significant changes in total cholesterol (TC) and high-density lipoprotein (HDL) levels. Major urinary protein 1 (Mup1), a key regulator of glucose and lipid metabolism (157), and energy expenditure (158) was significantly upregulated in liver and both WAT depots of *Fst*-Tg mice compared to the WT (148). Braga et al. previously reported a significant decrease in Mup1 gene expression in *Fst*-KO MEF differentiating cultures compared to the WT (144). In another recent study, Davey et al. reported that intravascular gene delivery *via* rAAV6-FST 317 to prediabetic *db/db* mice ameliorates progression of hyperglycemia, maintains insulinemia, promote abundance of insulin producing beta cell population, and reduced number of α‐like cells (159). The authors also reported that *Fst* gene delivery to older mice with hyperglycemia and declining insulinemia led to significant restoration of serum insulin concentration. Diabetic *db/db* mice display compromised β-cell function and reduced insulin content. Overexpression of FST in pancreatic-β cells has previously been reported to counter insulin insufficiency and extend the life span of *db/db* mice mainly by inhibition of SMAD pathway and activation of the PI3-kinase/Akt pathway (160). Most recently, Tang et al. also reported significant decrease in body fat percentage in mice on normal diet, and ameliorated the increase in body fat after HFD following AAV-Fst mediated gene delivery (145). In this study, *Fst* gene delivery in the HFD group significantly decreased serum levels of insulin, leptin, resistin, and C-peptide as well as serum glucose, triglycerides, cholesterol, and free FFAs as compared to control group. AAV-*Fst* gene delivery also significantly increased circulating levels of vascular endothelial growth factor (VEGF) and lowered serum levels of inflammatory cytokine IL-1α. In addition, reduced levels of mitochondrial oxidative phosphorylation (OXPHOS) complex subunits in subcutaneous WAT of mice on HFD was normalized following Fst overexpression *via* increased expression of PGC-1α. Although Fst has been reported to promote PGC-1α expression in previous studies (144, 147), the precise mechanism responsible for Fst-induced upregulation of PGC-1α remains unknown. Collectively, these findings provide exciting supporting evidence that Fst gene therapy could elicit beneficial metabolic effects and mitigate HFD-induced obesity. In order to test the effect of FST overexpression on overall lipidomic profiles in differentiating 3T3-L1 cells, Singh et al. performed comparative metabolic profiling of basal 3T3-L1 and Fst overexpressing 3T3-L1 *Fst* cells (148). Increased mitochondrial biogenesis in differentiated 3T3-L1 *Fst* cultures was also confirmed by significantly increased maximal oxygen consumption rate (OCR) (148). Analysis of endogenous lipid metabolites displayed a general reduction in diglycerides (DG), triglycerides (TG), ceramide, FA, phosphatidylcholine (PC), phosphatidylethanolamine (PE), and lysophosphatidylethanolamine (LPE) in FST overexpressing 3T3-L1 Fst cells compared to the basal 3T3-L1 cells (148). On the other hand, levels of several lyosophosphatidylcholines (LPL) such as LPC (16.0), LPC (18.0), and LPC (18.1) were significantly increased in 3T3-L1 *Fst* cells in comparison with the 3T3-L1 cells (148). These *in- vitro* data, thus, provide supporting evidence that genetic manipulation of FST could favorably alter overall lipid metabolites known to be associated with fat mass and promote obesity and associated metabolic conditions (161, 162). *In-vivo* analysis of adipose tissues from *Fst*-Tg mice also show significant differences in several amino acids including leucine, isoleucine, and valine also collectively referred to as branched-chain amino acids (BCAA), key components of urea cycle and arginine metabolism, and components of the Kreb’s cycle including citrate, succinylcarnitine, and fumarate were significantly lower compared to the WT tissues. FST overexpression in Fst-Tg mice was associated with significant upregulation of two key BCAA catabolic proteins BCAT2 and BCKDHA in epididymal WAT (148). Several recent studies have provided convincing evidence in support of a positive association between BCAA levels and insulin resistance and type 2 diabetes as their levels are significantly induced in obese subjects compared to the lean humans (163, 164). Levels of ω-3 polyunsaturated fatty acids (PUFAs), reported to improve obesity-associated chronic inflammation, insulin resistance and dyslipidemia (165), and regulate several aspects of energy and lipid metabolism (166) were significantly increased in the subcutaneous WAT of Fst-Tg mice. Levels of key lysolipids, known metabolic regulators of childhood obesity (167) were significantly elevated in the Epi WAT of *Fst*-Tg compared to the WT mice. Combined together, these findings obtained from comprehensive metabolomic profiling of Fst transgenic mice provide compelling evidence that genetic manipulation of *Fst in-vivo* favorably alters the levels of key metabolites known to influence various aspects of metabolic conditions, and warrant future studies for the use of FST based therapeutic interventions to combat obesity and related diseases (168).

## Conclusion

Obesity and associated comorbidities resulting from accumulation of dysfunctional white adipose tissues and chronic imbalance between energy intake and energy expenditure represent a growing worldwide problem. Activation of adipose browning characteristics leads to the dissipation of excess stored energy and provide metabolic benefits to combat the burden of obesity and related abnormalities including insulin resistance, hyperlipidemia, type 2 diabetes and cardiovascular diseases. Adipose browning phenomenon in humans has been confirmed based on both morphological, and functional studies (5, 6, 16). Accordingly, new strategies are being explored to identify novel compounds that can promote adipose browning and reduce the development of obesity and associated conditions. Recent reports from several laboratories provide convincing evidence that inhibition of TGF-β signaling pathway provides metabolic protection from obesity and diabetes by regulating glucose and energy homeostasis *via* activation of white adipose browning (13, 59). Genetic inactivation of MST, a key member of TGF-β superfamily not only results in increased muscle mass but also promotes activation of adipose browning and favorably alters several metabolic parameters implicated in the development of metabolic complications (86, 87). Since Fst is a known inhibitor of MST and reported to antagonize overall TGF-β signaling, it is logical to explore the therapeutic potential of FST in regulating key metabolic functions in both adipose depots besides its established role in promoting muscle mass. Recent findings by Braga et al. provided the first evidence that FST enhances the acquisition of beige and brown adipose characteristics by directly targeting Myf5- and Myf5+ populations to promote beige and brown adipose characteristics respectively (142). Since Myf5+ precursor population gives rise to both skeletal muscle and brown fat (20), it is not surprising that FST could selectively target these populations to promote both muscle and BAT mass (147, 169). Additionally, identification of key molecular and cellular targets responsible for FST-induced adipose browning is necessary to develop therapeutic strategies for the treatment of obesity and related diseases. Although activation of p38MAPK and ERK1/2 signaling is necessary for FST-induced adipose browning in both adipose depots (147), it is important to explore the possible role of irisin/Fndc5 during the process as secretion of irisin and subsequent activation of p38 MAPK and ERK1/2 has been reported during exercise (94). Since FST secretion is also induced following exercise (169, 170) and rFST treatment leads to elevated Fndc5 gene expression in muscle (86), it is possible that FST will indirectly affect p38MAPK and ERK1/2 activation *via* increased secretion of irisin. β3-AR signaling has been shown to promote p38 MAPK activation and induce browning of WAT and nonshivering thermogenesis in BAT (150, 171, 172). It is, therefore, possible that FST activates β3-AR signaling to promote p38 MAPK phosphorylation during adipose browning as β3 agonist CL 316,243 treatment elicited additive response in UCP1 levels in both WAT depots as well as in BAT (147). FGF21, another key regulator of adipose browning and a downstream target of β3-AR signaling (173, 174) is upregulated in WAT of Fst transgenic mice, suggesting a possible link between FST and FGF21 signaling during adipose browning. Based on available data, it appears that the beneficial effects of FST on adipose browning, obesity, and related metabolic conditions are mainly due to blocking of TGF-β ligands including MST and inhibition of Smad3 signaling as summarized in **Figure 1**. Finally, data obtained from Fst gene therapy studies in both human and nonhuman primates did not indicate apparent structural or functional aberration in various tissues, suggesting that FST may have therapeutic potential in clinical settings for the treatment of obesity and related diseases.

## Author Contributions

RS and SP organized and wrote the manuscript. SR edited the manuscript and provided constructive comments. All authors contributed to the article and approved the submitted version.

## Funding

This work was supported by National Institute of Health grant numbers SC1AG049682 (RS), SC1CA232319 (SP), TRDRP grant number T31IP1551 (RS, SR), Boston Pepper Center P30 AG031679 (RS), UHI NIMHD S21MD000103, and Accelerating Excellence in Translational Sciences (AXIS) Center U54MD007598 to Charles R. Drew University of Medicine and Science.

## Conflict of Interest

The authors declare that the research was conducted in the absence of any commercial or financial relationships that could be construed as a potential conflict of interest.
